# Molecular Mechanisms That Influence the Macrophage M1–M2 Polarization Balance

**DOI:** 10.3389/fimmu.2014.00614

**Published:** 2014-11-28

**Authors:** Nan Wang, Hongwei Liang, Ke Zen

**Affiliations:** ^1^State Key Laboratory of Pharmaceutical Biotechnology, Nanjing University School of Life Sciences, Nanjing, China; ^2^Jiangsu Engineering Research Center for MicroRNA Biology and Biotechnology (JERC-MBB), Nanjing University School of Life Sciences, Nanjing, China

**Keywords:** innate immune response, macrophage polarization, TLR, NLR, SOCS, microRNA

## Abstract

As an essential component of innate immunity, macrophages have multiple functions in both inhibiting or promoting cell proliferation and tissue repair. Diversity and plasticity are hallmarks of macrophages. Classical M1 and alternative M2 activation of macrophages, mirroring the Th1–Th2 polarization of T cells, represent two extremes of a dynamic changing state of macrophage activation. M1-type macrophages release cytokines that inhibit the proliferation of surrounding cells and damage contiguous tissue, and M2-type macrophages release cytokines that promote the proliferation of contiguous cells and tissue repair. M1–M2 polarization of macrophage is a tightly controlled process entailing a set of signaling pathways, transcriptional and posttranscriptional regulatory networks. An imbalance of macrophage M1–M2 polarization is often associated with various diseases or inflammatory conditions. Therefore, identification of the molecules associated with the dynamic changes of macrophage polarization and understanding their interactions is crucial for elucidating the molecular basis of disease progression and designing novel macrophage-mediated therapeutic strategies.

## Introduction

As an essential component of innate immunity, macrophages are capable of differentiating into protean varieties with a range of function (1–3). In respond to various environmental cues (e.g., microbial products, damaged cells, activated lymphocytes) or under different pathophysiologic conditions, macrophages can acquire distinct functional phenotypes via undergoing different phenotypic polarization (4). Macrophage M1 and M2-type responses describe the opposing activities of killing or repairing, and such polarized responses stimulate Th1- or Th2-like responses in macrophages, respectively. First, M1 phenotype is stimulated by microbial products or pro-inflammatory cytokines [IFN-γ, TNF, or Toll-like receptor (TLR) ligands], and the typical characteristics of M1 macrophages include high antigen presentation, high production of IL-12 and IL-23, and high production of nitric oxide (NO) and reactive oxygen intermediates (ROI) (5). In contrast, M2-type responses are the “resting” phenotype and are observed in healing-type circumstances without infections. Such responses can also be further amplified by IL-4, IL-10, or IL-13. M2 macrophages are characterized by the upregulation of Dectin-1, DC-SIGN, mannose receptor, scavenger receptor A, scavenger receptor B-1, CD163, CCR2, CXCR1, and CXCR2 (6). Instead of generating NO or ROI, M2 macrophages produce ornithine and polyamines through the arginase pathway (2, 7). In fact, from the functional point view, NO and Ornithine, correlating to killing (M1) and repairing function (M2) of macrophages, have been regarded by some investigators as the most characteristic molecules of macrophages (8). Second, inflammatory M1 macrophages produce many other pro-inflammatory cytokines like TNFα, IL-1, IL-6, IL-12, Type I IFN, CXCL1-3, CXCL-5, and CXCL8-10 (9), while M2 macrophages generate anti-inflammatory cytokine such as IL-10 and very low level of pro-inflammatory cytokine such as IL-12 (10). Additional signatures of M2 phenotype, such as YM1 (a member of the chitinase family) and FIZZ1 (found in inflammatory zone 1, RETNLA) are also identified (11). Third, M1 macrophages promote Th1 response and possess strong microbicidal and tumoricidal activity, while M2 macrophages are involved in metazoan parasites containment and promotion of Th2 response, tissue remodeling, immune tolerance, and tumor progression (12, 13). Additional information about polarized activation of macrophages can be found in the previous reviews (1, 14–16).

A coordinate action of various inflammatory modulators, signaling molecules, and transcription factors is involved in regulating macrophage polarization. At cellular level, although M1 and M2 macrophage activities exist without T or B cell influence (17), specialized or polarized T cells (Th1, Th2, Tregs) do play a role in macrophage polarized activation (1). Canonical IRF/STAT signaling is a central pathway in modulating macrophage polarization. Activation of IRF/STAT signaling pathways by IFNs and TLR signaling will skew macrophage function toward the M1 phenotype (via STAT1), while activation of IRF/STAT (via STAT6) signaling pathways by IL-4 and IL-13 will skew macrophage function toward the M2 phenotype (9). Signals initiated by IL-10, glucocorticoid hormones, apoptotic cell-released molecules, and immune complexes can also profoundly affect macrophage functional statue (1). Macrophage polarization is also modulated by local microenvironmental conditions such as hypoxia (18). More importantly, M1–M2 polarization of macrophage is a highly dynamic process and the phenotype of polarized macrophages can be reversed under physiological and pathological conditions (19, 20). In the course of various pathophysiological settings, the same signaling pathway can be involved in either M1 or M2 polarization of macrophages. The molecular mechanisms that govern the phenotype switch of macrophages, however, remains incompletely understood. Moreover, imbalances of macrophage M1–M2 polarization are associated with various diseases. Disease conditions are frequently associated with polarization of macrophage activation, with classically activated M1 macrophages implicated in initiating and sustaining inflammation and M2 macrophages associated with resolution of chronic inflammation (6). In the past decade, a new class of small non-coding RNAs, termed as microRNAs (miRNAs), have emerged as important regulators in biological processes. An important role of miRNAs in modulating macrophage phenotypic polarization is demonstrated by accumulating evidences in which an excessive or impaired inflammatory response of macrophages is found to be tightly linked to the deregulation of miRNAs. In this review, we focus on recent progress in understanding the molecular basis underlying the dynamic macrophage polarization, including signaling pathways, transcription factors and miRNAs.

## IRF/STAT Signaling

**Figure 1:**
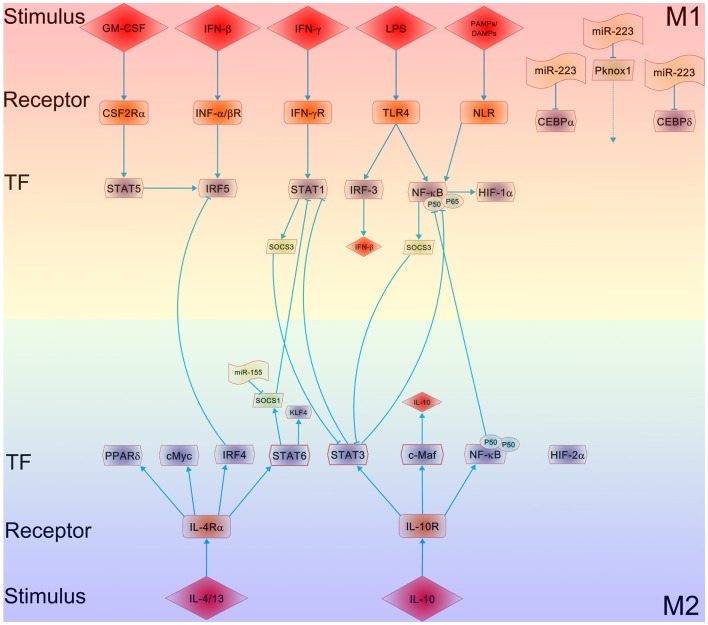
**Mechanisms underlying the polarization of macrophages**. The major regulatory pathways of macrophage M1–M2 polarization are outlined. The crosstalk between the M1 and M2 macrophage polarizing pathways, particularly the balance between activation of STAT1 and STAT3/STAT6, tightly regulates macrophage polarization and activity. A predominance of NF-κB and STAT1 activation promotes M1 macrophage polarization, resulting in cytotoxic and tissue-damage proinflammatory functions. In contrast, a predominance of STAT3 and STAT6 activation by IL-4/13 and IL-10 increases M2 macrophage polarization, associated with immune tolerance and tissue repairing. PPARδ (and PPARγ) control distinct aspects of M2 macrophage activation and oxidative metabolism. KLF-4, a downstream of STAT6, participates in the promotion of M2 macrophage functions by suppressing the NF-κB/HIF-1α-dependent transcription. IL-4 induces not only c-Myc, which controls the expression of a subset of M2-associated genes but also the M2-polarizing IRF-4 axis to inhibit IRF5-mediated M1 polarization. IL-10 promotes M2 polarization through the induction of p50 NF-κB homodimer, c-Maf, and STAT3 activities. MicroRNAs such as miR-155, miR-223, etc. are involved in modulating macrophage polarization via targeting SOCS1, CEBP, and Pknox1, respectively.

As shown in Figure 1, IRF/STAT signaling is a central pathway in controlling macrophage M1–M2 polarization. Toll-like receptor signaling, particularly TLR4 stimulated by lipopolysaccharide (LPS) and other microbial ligands, drives macrophages to a preferentially M1 phenotype. Two adaptors, MyD88 and TRIF, mediate the signaling downstream of TLR4. The signaling pathway through the MyD88 adaptor results in the activation of a cascade of kinases, including IRAK4, TRAF6, and IKKβ, which finally leads to the activation of nuclear factor kappa B (NF-κB). As a key transcription factor related to macrophage M1 activation, NF-κB regulates the expression of a large number of inflammatory genes including TNFα, IL1B, cyclooxygenase 2 (COX2), IL-6, and IL12p40. NF-κB activity is modulated via the activation of the inhibitor of kappa B kinase (IKK) trimeric complex (two kinases, IKKα, IKKβ, and a regulatory protein, IKKγ). When upstream signals converge at the IKK complex, they first activate IKKβ via phosphorylation, and activated IKKβ further phosphorylates the inhibitory molecule, inhibitor of kappa B (I-κB). This results in the proteosomal degradation of I-κB and the release of NF-κB p65/p50 heterodimer from the NF-κB/I-κB complex. The NF-κB p65/p50 heterodimer is then translocated to the nucleus and binds to the promoters of inflammatory genes. The signaling through the TRIF adaptor pathway activates the transcription factor interferon-responsive factor 3 (IRF3), leading to the expression and secretion of type I interferon, such as IFNα and IFNβ. Secreted type 1 interferons bind to the type I interferon receptor (IFNAR) with consequent activation of the transcription factor STAT1. It has been widely reported that IRF3 and IRF5 are involved in regulating M1 polarization and M1-associated gene induction (21, 22). IFN-stimulated genes include chemokine CXCL9 and CXCL10 (23), which are characteristic of classical M1 macrophage activation. In fact, macrophage polarization is tightly linked to the differential expression of various TLRs on macrophages. The ratio of TLR4/TLR2 is significantly higher in M1 macrophages compared to M2 macrophages (24), while TLR4 deficiency promotes the alternative activation (M2) of adipose tissue macrophages (ATMs) (25). TLR ligands, e.g., imiquimod and CpG, have been used as therapeutic treatments for inflammatory diseases such as asthma by modulating macrophage polarization.

Toll-like receptor and Toll-like receptor-induced cytokine-receptor cascades are broadly inhibited by tyrosine kinases Tyro3, Axl, and Mer. IFNβ can activate the receptor for Axl, Tyro3, and Mer and negatively regulate TLR signaling through induction of SOCS1 and SOCS3 (26). A hyperactive signaling mediated by Tyro3, Axl, and Mer receptor is suggested to induce immunosuppression in severe sepsis patients (26). Along the same lines, chronic signaling through the TLR4 pathway has been shown to induce various negative regulators like IRAK-M, ST2, SOCS1, short version of MyD88 (MyD88sh) (27, 28) and SHIPs (29). These negative regulators inhibit TLR-mediated signaling and thus switch macrophages to an immunosuppressive, endotoxin-tolerant phenotype. A switching from an MyD88-dependent to a TRIF-dependent TLR4 pathway in macrophages has also been suggested to shift macrophage phenotype from an inflammatory to anti-inflammatory, endotoxin-tolerant phenotype (30). Thus, interplay of signaling molecules and transcription factors can reverse the phenotype of macrophage polarization.

STAT-mediated activation of macrophages is regulated by members of the suppressor of cytokine signaling (SOCS) family. SOCS family members are inducible inhibitors of cytokine signals and thus play a critical role in limiting inflammation responses. SOCS proteins could be induced by cytokine signaling pathway, and they in turn inhibit the cytokine signaling by several mechanisms. For example, IL-4 and IFN-γ, the latter in concert with TLR stimulation, upregulate SOCS1 (31) and SOCS3 (32), which in turn, inhibit the action of STAT1 and STAT3, respectively. SOCS proteins can be also directly induced by TLR signaling. In macrophages, SOCS proteins not only regulate the sensitivity of cells toward cytokines but also modulate signaling through TLRs. Because SOCS3 is a downstream molecule of Notch signaling (33), it is likely that Notch signaling determines the M1 versus M2 polarization of macrophages through SOCS3 (34). However, the role of SOCS3 in modulating macrophage M1–M2 polarization is controversial. Although the unique expression of SOCS3 was reported to be essential for classic macrophage activation (32), SOCS3 deficiency also promotes M1 macrophage polarization and inflammation (35).

Macrophages can be driven to M2 phenotype by canonical M2 stimuli like IL-4, IL-13, and IL-10 (36, 37). As shown in Figure 1, IL-4 and IL-13 polarize macrophages to M2 phenotype via activating STAT6 through the IL-4 receptor alpha (IL-4Rα), whereas IL-10 promotes M2 phenotype via activating STAT3 through receptor (IL-10R). In IL-4 and IL-13 pathway, receptor binding of IL-4 activates JAK1 and JAK3 (38), leading to STAT6 activation and translocation. Macrophage M2 phenotype is promoted by several transcription factors, including peroxisome proliferator activated receptor γ (PPARγ) (39, 40) and Krueppel-like factor 4 (KLF-4) (41). Myeloid-specific deficiency of either PPARγ or KLF-4 resulted in suppressed M2 polarization of macrophages, leading to accelerated lesion formation in apolipoprotein E-deficient (42) or low-density lipoprotein receptor-knockout (43) mice. Moreover, ligation of PPARγ by specific PPARγ ligands resulted in a preferential M2 polarization in mice and in human beings (40). Other transcription factors involved in this process include c-Myc and IRF4. Transciptome analysis of IL-4-stimulated cells consists of various enzymes and transcription factors, including transglutaminase 2 (TGM2), mannose receptor, cholesterol hydroxylase CH25H, prostaglandin-endoperoxide synthase PTGS1 (prostaglandin G/H synthase 1), transcription factors IRF4, KLF-4, and the signaling modulators CISH and SOCS1 (44). During severe respiratory syncytial virus (RSV)-induced bronchiolitis, IL-4Rα/STAT6-dependent M2 differentiation of macrophages reduces inflammation and epithelial damage in lungs (45).

PPARγ play an important role in modulating macrophage M2 polarization induced by IL-4 or IL-13 (46). Studies using PPARγ-deficient macrophages have shown the role of this nuclear receptor in promoting M2 activation to protect mice from insulin resistance (47). A similar role was also found for the PPARδ in determination of macrophage polarization (48). Using myeloid-specific transcription factor KLF-4 knockout mice, Liao et al. (41) demonstrated the role of KLF-4 in regulating M2 polarization of macrophages as well as in protecting mice from obesity-induced insulin resistance. In a similar fashion, IRF4 is also involved in regulating the expression of genes associated with macrophage M2 polarization (49). Collectively, all these findings suggest that STAT6, PPARγ, KLF-4, and IRF4 may coordinate the M2 polarization of macrophages.

IL-10R, a heterodimer of IL-10R1 and IL-10R2, is a receptor for IL-10. Ligation of IL-10R with IL-10 results in the autophosphorylation of IL-10R, leading to the activation of the transcription factor STAT3 and reduction of pro-inflammatory cytokine expression. In macrophages, IL-10 is also reported to respond to TLR activation, glucocorticoid treatment, and C-type lectin signaling (e.g., DC-SIGN and dectin 1 ligation). The components in IL-10-induced macrophage transcriptome include specific Fc receptors, chemoattractants CXCL13 and CXCL4, recognition receptors formyl peptide receptor 1 (FPR1), TLR1, TLR8, and macrophage receptor with collagenous domain (50).

## HIF-1α and HIF-2α

Macrophages can rapidly alter their metabolic and functional state to adapt to the microenvironment of surrounding tissues. Microenvironmental conditions in infected, inflamed, or damaged tissues are generally lack of oxygen and nutrients. When macrophages are recruited into inflammatory sites, they encounter the hypoxia condition, which can directly affect the macrophage polarization. Hypoxia executes its effect on macrophages through two isoforms of hypoxia-inducible factor (HIF), HIF-1α and HIF2 (51, 52). Gene expression profiling of macrophages and monocytes has identified profound changes in response to hypoxia (53, 54). Hypoxia strongly induces the expression of angiogenesis- and metastasis-related genes such as VEGF, FGF2, MMP7, and MMP9. Upregulation of those genes under hypoxia would lead to more recruitment of macrophages into the hypoxic (avascular) areas in pathologies like atherosclerosis, obesity, and cancer where they dampen the inflammation or promote tumor progression. In addition, pro-inflammatory cytokines like TNFα, IL-1β, MIF, CCL3, and COX2, as well as M2 markers like IL-10 and arginase 1 in macrophages, are also induced by hypoxia (55). The crucial role of hypoxia in regulating macrophage inflammatory response has been confirmed in mice with myeloid cell-specific deletion of HIF-1α (56), in which HIF-1α was found to be essential in regulating myeloid cell glycolytic capacity and survival and function in the inflammatory microenvironment. This is in line with the finding that HIF-1α was induced by NF-κB (57) and plays an important role in modulating macrophage phagocytosis of bacteria under sepsis conditions (58). Moreover, recent studies also showed that HIF-1α can mediate the effects of tumor-derived lactic acid (59) and cytokines (Oncostatin M and Eotaxin) (60) on promoting M2-like phenotype. In contrast to these studies, recent study of myeloid-specific HIF-2α deletion showed the role of HIF2 in mediating macrophage inflammatory responses rather than HIF-1α (52). In contrast to these studies, a recent (61) suggested that HIF-1α and HIF-2α might also drive macrophage polarization by modulating NO homeostasis in a cytokine-induced and transcription-dependent fashion. Specifically, this study showed that inducible NO synthase gene and the arginase 1 gene in polarized macrophages are specifically regulated by HIFs (61). Although HIF-1α and HIF-2α displayed physiologically antagonistic functions, their antiphase regulation allows them to coordinately regulate NO production to guide macrophage polarization. Together, these findings suggest HIFs as an important regulator of macrophage polarization, although a detailed dissection of whether the alteration of HIF isoform expression can switch macrophage phenotypes needs further investigation.

## Oligomerization Domain (NOD)-Like Receptors

Stimulated by a diverse set of stimulus, including interferon-γ (IFN-γ), LPS, and other TLR activators, macrophages are polarized toward to M1 state in which oxidative metabolites and pro-inflammatory cytokines are produced. Engagement of the respective receptors by these stimulus results in activation of the adapter proteins such as MyD88, leading to sequential activation of kinases, phosphorylation of transcription factors, and eventual genetic program induction. Pro-inflammatory genes, including IFN-γ, tumor necrosis factor-α (TNF-α), IL-1β, IL-18, chemokines, and proteases, are subsequently produced. Further activation of the M1 pathway occurs through the assembly of the NLR inflammasome and caspase-1 activation, which results in the conversion of IL-1β and IL-18 into secreted active forms (62). With the NLRP3 inflammasome serving as a sensor of obesity-associated danger signals, the progression of obesity can switch macrophages from “M2-like” to “M1-like” cells (63). In macrophages, the activation of NLR stimulates the cryptopyrin/NLRP3 inflammasome to induce IL-1β and IL-18 production via caspase-1. Caspase-1 and IL-1β are induced in adipose tissue with diet-induced obesity (DIO), and Nlrp3- and caspase-1-deficient mice both demonstrate a resistance to DIO-induced inflammation (63). The mechanism of this protective effect may be driven by the alteration of M1 activation of ATMs, as Nlrp3-knockout mice show decreased M1 but increased M2 gene expression in ATMs.

In addition to binding to TLRs, some pathogen-associated molecular patterns (PAMPs) are also recognized by a family of cytosolic nucleotide-binding receptors and NOD-like receptors (NLRs) (64), another groups of PAMP receptors. Some NLRs are involved in the recognition of microbial molecules and/or endogenous factors released from tissue destruction. This recognition can lead to activation of caspase-1 (a pro-inflammatory caspase), and subsequent proteolytic conversion of potent pro-inflammatory cytokines IL-1β and IL-18 from their precursors pro-IL-1β and pro-IL-18, respectively. The proteolytic conversion of IL-1β and IL-18 is mediated by a cytosolic caspase 1-activating protein complex, termed as inflammasome (65).

As the most well-characterized members of the NLR family, NOD1 is ubiquitously expressed and NOD2 is restricted to monocytes, macrophages, dendritic cells, and intestinal Paneth cells (66). Both NOD1 and NOD2 induce NF-κB activation in a TLR-independent manner (67). Structural analysis demonstrated that NOD1 and NOD2 recognize different core motifs derived from peptidoglycan (PGN), a component of bacterial cell walls. NOD1 activity is triggered by γ-d-glutamyl-meso-diaminopimelic acid, a unique PGN structures from all Gram-negative and some Gram-positive bacteria (68). In contrast, NOD2 is activated by muramyl dipeptide, a PGN motif in all Gram-positive and Gram-negative bacteria (69). Upon ligand recognition, NOD1 and NOD2 undergo conformational changes and self-oligomerization, which is followed by the recruitment and activation of the serine threonine kinase RICK (RIP2, also known as RIPK2), an essential step for the activation of NF-κB and MAPKs. The ubiquitination of RICK is essential for NOD1/NOD2-mediated signaling because removal of this modification by deubiquitinating enzyme A20 largely dampens NOD1/NOD2-induced NF-κB activation (70, 71). Although both NOD1 and NOD2 induce similar K63-linked ubiquitination of RICK for NF-κB activation and upregulation of various inflammatory mediators, NOD2 signaling appears to preferentially utilize the E3 ligase TRAF6 and NOD1-mediated signaling is mainly associated with TRAF2 and TRAF5. Nevertheless, the role of NOD1 and NOD2 in activating NF-κB-dependent inflammatory responses is not limited to the recognition of PGN motifs. Recent study by Keestra et al. (72) reported that that NOD1 can sense activation state of small Rho GTPases. In this study, NOD1 signaling pathway was triggered by RAC1 and CDC42 activated by bacterial delivery or ectopic expression of SopE, a virulence factor of the enteric pathogen *Salmonella*.

## Granulocyte-Macrophage Colony-Stimulating Factor

As the most recently discovered cytokine involved in regulation of macrophage polarization, granulocyte-macrophage colony-stimulating factor (GM-CSF) is produced by a variety of cells including macrophages and parenchyma cells. The main functions of GM-CSF include regulating the proliferation and differentiation of functional hematopoietic cells. The GM-CSF receptor forms a dodecamer structure (73) and recruits JAK2, leading to the activation of STAT5, extracellular signal-regulated kinase (ERK), V-Akt murine thymoma viral oncogene homolog 1 (AKT), and the nuclear translocation of NF-κB and IRF5 (21). Many of these regulators are part of the IFN-γ and TLR signaling pathways. GM-CSF enhances macrophage antigen presentation, complement- and antibody-mediated phagocytosis, microbicidal capacity, leukocyte chemotaxis, and adhesion. GM-CSF induces cytokine production of IL-6, IL-8, G-CSF, M-CSF, TNF, and IL-1β in monocytes and macrophages, although the degree of cytokine induction by GM-CSF is less than that by LPS. Global gene expression analyses of macrophages differentiated from GM-CSF-treated monocytes showed that GM-CSF upregulated 340 genes and downregulated 190 genes in macrophages. Macrophage-specific genes including CD14, CD163, C5R1, and FcγR1A, and several cell surface adhesion molecules, cytokine receptors were induced by GM-CSF (74). In this study, a high-resolution transcriptome profiling of human macrophages by RNA sequencing was employed to discover novel marker genes unique for human macrophages. A similar strategy has been used to obtain a high-resolution transcriptome profile of human macrophages under M1 (or M1-like) and M2 (or M2-like) polarization conditions, resulting in a more comprehensive understanding of the transcriptome of human macrophages (75). The GM-CSF deficient mice have normal numbers of macrophages in many tissues but display an impaired maturation of alveolar macrophages and develop pulmonary alveolar proteinosis (76). In human beings, mutations in the GM-CSF receptor, especially in the common beta chain, lead to alveolar macrophage dysfunction, proteinosis, and malignancy (77, 78).

## MicroRNAs

MicroRNAs (miRNAs), a class of 19–24 nt non-coding RNAs that induce gene silencing at the posttranscriptional level, have emerged as an important regulatory mechanism for gene expression in many immune cells including monocytes and macrophages (79, 80). Functional miRNAs associated with polarized macrophages have been identified (81). While these functional miRNAs like miR-155 and miR-146 are induced by a variety of inflammatory stimuli, including LPS, TNFα, and IL-1β, they are instrumental in attenuating TLR4/IL-1R signaling pathways in monocytes and macrophages (79, 82, 83). These findings allow us to postulate that miRNAs may contribute to the switching of inflammatory macrophages to an immunosuppressive phenotype, needed for resolution. For instance, miR-146, miR-125b, miR-155, and miR-9 have been shown to be induced by LPS, and in turn, these miRNAs inhibit TLR4/IL-1R signaling through regulation of IRAK-1, TRAF6, IKKe, p50NF-jB, and TNFα at transcriptional and posttranscriptional level (79, 82–86). Our recent study has also shown that a panel of miRNAs including miR-17, miR-20a, and miR-106a are stimulated by LPS through c-Myc pathway, and these miRNAs collectively reduces the expression level of macrophage differentiation related marker, signal-regulatory protein α (SIRPα) (87). It has been reported that miR-98 and miR-21 inhibit the expression of inflammatory genes in monocytes and macrophages via controlling IL-10 level (88, 89). These findings strongly argue that miRNAs can regulate macrophage phenotype in the course of various diseases, for example, during endotoxin tolerance (30, 90).

In the efforts to delineate the role of miRNAs in macrophage activation in inflammatory diseases, Ponomarev et al. (91) found that miR-124 promotes microglia quiescence and suppresses EAE by deactivating macrophages in a C/EBPα-PU.1-dependent manner. This is one of the few studies in which a specific miRNA is found to regulate macrophage plasticity, although it remains unclear how C/EBPα suppresses macrophage M2 polarization. Zhang and co-workers (92) also reported that miR-223 modulates obesity-associated adipose tissue inflammation through regulating macrophage activation. In the study, they found that miR-223 was upregulated in LPS-treated macrophages but downregulated in IL-4-treated bone marrow-derived macrophages (BMDMs). In agreement with the observation of differential expression of miR-223 in various macrophages, the miR-223-deficient macrophages were hypersensitive to LPS stimulation, whereas such macrophages exhibited a delayed responses to IL-4 compared with controls. Moreover, miR-223-deficient mice exhibited an increased adipose tissue inflammatory response but a decreased adipose tissue insulin signaling. They further identified Pknox1 as a genuine target of miR-223. Although Pknox1 as a miR-223 target in regulating macrophage polarization was validated by gain-of-function and loss-of-function analyses in BMDMs, it remains unclear how Pknox1 further regulates macrophage polarization.

A recent work by Banerjee et al. (93) demonstrated that let-7c could regulate bactericidal and phagocytic activities of macrophages, two functional phenotypes implicated in macrophage polarization. In the study, they found that let-7c was expressed at a higher level in M2-type macrophages than in M1-type macrophages. When M2-type macrophages were re-polarized to M1-type macrophages or M1-type macrophages converted to M2-type macrophages, let-7c expression level was decreased or increased, respectively. As LPS stimulation reduced let-7c expression in M2 macrophages, let-7c might play an inhibitory role in modulating macrophage inflammatory responses. In line with this, upregulation of let-7c in macrophages diminished M1 phenotype but promoted M2 phenotype polarization. Their study further identified that let-7c targeted C/EBP-δ, a key transcriptional factor in macrophage pro-inflammatory response to TLR4 stimulation (94, 95).

The modulation of macrophage polarization by miR-155 has also been recently reported (96, 97). The expression of miR-155 was found to be repressed in naive macrophages or LPS-stimulated Akt2^-/-^ macrophages. In this process, miR-155 targets transcriptional factor C/EBP-β, a hallmark of M2 macrophages. C/EBP-β can regulate Arg1 and its level is increased upon Akt2 ablation. Overexpression or depletion of miR-155 drove macrophages to M1 or M2 phenotype, respectively, confirming that miR-155 plays a central role in regulating Akt-dependent M1/M2 polarization of macrophages. It has also reported that miR-155 can directly block IL-13-induced macrophage M2 phenotype via suppressing the expression of IL-13Rα1 (96). As an oncomiR, miR-155 also targets SHIP1 to promote TNFα-dependent tumor cell growth (98). Through overexpression of miR-155, we successfully re-polarized tumor-associated macrophages (TAMs) into pro-inflammatory M1 macrophages (97). Taken together, these studies support the hypothesis that miR-155 is a key molecule in causing macrophage polarization toward M1-type activity.

## M1–M2 Phenotype Switch

Macrophage differentiation is highly dynamic. Responding to microenviromental cues macrophages can rapidly switch from one phenotype to the other. In fact, activation of NF-κB or IRF family members in macrophages by TLR4 or other TLRs can drive macrophage to either M1 or M2 polarization under various pathological conditions (99–105). Accumulating evidences have shown that the spatiotemporal activation of NF-κB is a key regulator of the plasticity of macrophages observed in the courses of various disease progressions. For example, during the early phase of tumorigenesis, NF-κB activation in M1 macrophages is critical for cancer-related inflammation. However, at the late phase of tumorigenesis, macrophages are re-programed to TAM or M2-like macrophages displaying low NF-κB activation but increased immunosuppressive capacity (106). A similar situation of macrophage polarization is observed at different stages along the progression of sepsis, in which NF-κB activation in M1 macrophages drives the initial overt inflammatory phase, while during the late phase of endotoxin tolerance, macrophages are polarized to an anti-inflammatory, tumor growth-promoting (M2) phenotype, and display an impaired NF-κB activation (107). The studies on RSV infection also show that polarization of macrophages is complicated process and the phenotype of macrophage activation can be varied at the different stage along disease progression. As the most significant cause of lower respiratory tract infection in infants and young children, RSV infection is found to be associated with a mixed “Th1” and “Th2” cytokine storm. At the initial stage of RSV infection, RAV induces the expression of various anti-viral genes like IFN-β in airway epithelial cells, and then promotes the expression of many NF-κB-dependent pro-inflammatory genes in macrophages through stimulating TLR4, TLR2, TLR3, and retinoic acid-induce gene I (RIG-I), driving macrophages toward anti-viral, pro-inflammatory M1 phenotype. However, to maintain a mild but persisting infection, RSV also induces alveolar macrophages to produce IL-4 and IL-13 that contribute to macrophage M2 polarization and disease resolution through IL-4Rα/STAT6-, TLR4-, and IFN-β-dependent signaling pathways (45).

Although under certain conditions like parasite infections and allergy, the functional phenotypes of macrophages *in vivo* largely mirror those of canonical M1 and M2-polarized states, macrophage populations often express mixed phenotypes in the course of various disease settings. Indeed, macrophages with combinations of M1 and M2 markers can be found in neurodegenerative disorders (108), atherosclerotic plaques (109), and some murine tumors (110). Therefore, the contribution of coexisting macrophages with different phenotypes, the impact of dynamic changes of macrophage plasticity on diseases, and the molecular networks orchestrating the switch of macrophage phenotype are required to be analyzed for a full understanding of the M1–M2 paradigm of macrophage polarization.

## Future Directions

Tremendous progress has been made in defining the molecular networks underlying M1–M2-polarized activation of macrophages. Molecular determinants of M1–M2 polarization include members of the PPAR, KLF, IRF, STAT, NF-κB, and HIF families, and miRNAs. However, new molecules that regulate macrophage M1–M2 polarization may still remain unidentified. A novel class of large intergenic non-coding RNAs, termed as lincRNAs, has been recently shown to be involved in both activation and repression of immune response genes (111). Among thousands of lincRNAs identified in the mammalian genome, 159 lincRNAs was found to be differentially expressed following innate activation of THP1 macrophages (112). In these differentially expressed lincRNAs, linc1992 was found to specifically bind to heterogenous nuclear ribonucleoprotein L (hnRNPL) and form a linc1992-hnRNPL complex that regulates TNFα gene transcription. The role of lincRNAs in modulation of macrophage polarization, however, has not been reported so far but certainly needs to be further studied.

Different from the irreversible phenotypic changes seen in lymphocytes after exposure to polarizing cytokines, macrophage polarization is transient and plastic. In order to adapt to the microenvironmental conditions of surrounding tissues, macrophages can rapidly switch their phenotypes. For example, M2 macrophages can be re-polarized into macrophages with M1 phenotype following exposure to TLR ligands or IFNγ or overexpression of miR-155 (113, 114), whereas M1 macrophages can be reprogramed to express various genes of M2 macrophage by treating macrophages with reagents that increase IL-10 level (115, 116). Therefore, further exploring the dynamic process of macrophage polarization and the mechanisms that govern this process not only is important for our understanding of the M1–M2 paradigm of macrophage polarization but also provides new therapeutic strategies for various diseases including cancers via targeting imbalances of macrophage polarization.

## Conflict of Interest Statement

The authors declare that the research was conducted in the absence of any commercial or financial relationships that could be construed as a potential conflict of interest.
